# Neutralization of antibody-enhanced dengue infection by VIS513, a pan serotype reactive monoclonal antibody targeting domain III of the dengue E protein

**DOI:** 10.1371/journal.pntd.0006209

**Published:** 2018-02-09

**Authors:** Yadunanda Budigi, Eugenia Z. Ong, Luke N. Robinson, Li Ching Ong, Kirk J. Rowley, Alexander Winnett, Hwee Cheng Tan, Sven Hobbie, Zachary Shriver, Gregory J. Babcock, Sylvie Alonso, Eng Eong Ooi

**Affiliations:** 1 Visterra Singapore International Pte Ltd, Singapore, Singapore; 2 Infectious Diseases Interdisciplinary Research Group, Singapore-MIT Alliance for Research and Technology, Singapore, Singapore; 3 Experimental Therapeutics Centre, Agency for Science, Technology and Research, Singapore, Singapore; 4 Program in Emerging Infectious Diseases, Duke-NUS Medical School, Singapore, Singapore; 5 Visterra Inc, Cambridge, Massachusetts, United States of America; 6 Department of Microbiology, Yong Loo Lin School of Medicine, National University of Singapore, Singapore, Singapore; University of North Carolina at Chapel Hill, UNITED STATES

## Abstract

Dengue virus (DENV) infection imposes enormous health and economic burden worldwide with no approved treatment. Several small molecules, including lovastatin, celgosivir, balapiravir and chloroquine have been tested for potential anti-dengue activity in clinical trials; none of these have demonstrated a protective effect. Recently, based on identification and characterization of cross-serotype neutralizing antibodies, there is increasing attention on the potential for dengue immunotherapy. Here, we tested the ability of VIS513, an engineered cross-neutralizing humanized antibody targeting the DENV E protein domain III, to overcome antibody-enhanced infection and high but brief viremia, which are commonly encountered in dengue patients, in various *in vitro* and *in vivo* models. We observed that VIS513 efficiently neutralizes DENV at clinically relevant viral loads or in the presence of enhancing levels of DENV immune sera. Single therapeutic administration of VIS513 in mouse models of primary infection or lethal secondary antibody-enhanced infection, reduces DENV titers and protects from lethal infection. Finally, VIS513 administration does not readily lead to resistance, either in cell culture systems or in animal models of dengue infection. The findings suggest that rapid viral reduction during acute DENV infection with a monoclonal antibody is feasible.

## Author summary

With currently no approved treatment options, dengue is a significant cause of hospitalizations and morbidity in the tropical and subtropical regions of the world. In recent years there is increased focus on the potential of antibody-based immunotherapy as treatment for dengue. In addition to being able to broadly neutralize all four serotypes of dengue, antibody-based therapeutics face additional challenges of interference with preexisting antibodies from the natural immune response, as well as possible viral resistance. In this study we demonstrate that VIS513, a monoclonal antibody therapeutic candidate that targets the dengue virus envelope protein, retains its anti-viral potency in the presence of preexisting antibodies, and is able to rescue mice from lethal infections. In addition, our studies indicate that VIS513 is generally refractory to emergence of viral resistance via mutations. Our data supports continued development of VIS513 as a treatment option for dengue.

## Introduction

Four serotypes of dengue virus (DENV1-4) are responsible for significant worldwide morbidity and mortality, with an estimated 390 million episodes of infection yearly [1]. Although infection with one serotype is thought to produce life-long immunity to the infecting serotype, secondary infection with a heterologous DENV serotype is associated with enhanced susceptibility to more severe disease through a mechanism that is not completely understood. Tertiary or quaternary infections appear to be less likely to elicit clinically overt disease although the number of studies that have been able to definitively document such infections is rare [2,3].

DENV contains two structural proteins on its surface, envelope (E) and precursor membrane (prM). The E protein is arranged as antiparallel homodimers on the surface of the mature virion, with a total of 180 E proteins covering the virion surface [4]. E protein is composed of three domains, DI-DIII, in which DII harbors the fusion loop required for viral entry [5]. The prM protein covers the fusion loop during virus assembly to prevent premature fusion, and is subsequently cleaved by a furin-like protease leading to dissociation of the precursor “pr” portion from the virus. However, the cleavage process is imperfect, leading to a range of virus structures from completely immature (no prM cleavage), to partially mature, to fully mature (complete prM cleavage) [6,7]. The resulting mosaic of virus structures influences epitope accessibility and thereby affects the neutralizing activity of different classes of antibodies [7,8].

Studies investigating the dominant types of neutralizing antibodies produced following full recovery from DENV infections have identified both homotypic and heterotypic antibody classes. Antibodies recognizing complex, quaternary epitopes appear to mediate a substantial fraction of the virus neutralizing activity in serum of convalescent individuals, however many of these antibodies are serotype-specific [9–11]. As such, this class of antibodies may help mediate homotypic protection after primary infection. The fusion loop epitope (FLE) region, located in DII, is another dominant neutralizing epitope region recognized upon DENV infection [12]. Antibodies to the fusion loop typically exhibit lower neutralizing activity, in part due to the more limited accessibility of this region, although they exhibit very broad neutralization reactivity to all four DENV serotypes [13]. More recent studies have indicated that after secondary infection, antibodies recognizing FLE exhibit enhanced avidity and neutralizing activity [14]. Another recently identified class of antibodies acquired following DENV infection recognizes the “envelope dimer epitope” (EDE), which is a region that localizes proximal to the FLE in DII [8]. Interestingly, this epitope is masked by prM on immature viral particles [8] and hence antibodies that target this epitope would not enhance infection of immature DENV by Fc receptor-mediated uptake [15]. However, such antibodies would also not prevent possible enhancement of immature DENV through anti-prM antibodies, which develop following primary dengue infection and form a major component of the antibody response during secondary dengue infection [16].

Antibodies to DIII often exhibit high neutralization potency and range in their breadth of reactivity from serotype-specific to cross-serotype-reactive, but rarely efficiently neutralize all four DENV serotypes [17]. Although antibodies targeting DIII are potent components of the humoral response to DENV infection in mice [18–20], studies from human samples have shown DIII-antibodies to have a minor role in neutralizing activity present in dengue-immune human sera [21–23]. This has also been reported for other flaviviruses, where very little antibody response to DIII was observed after tick-borne encephalitis virus (TBEV) infection or yellow fever virus (YFV) vaccination [24,25]. We recently described an engineered, humanized antibody, VIS513, which binds an exposed region in DIII which is strongly neutralizing for all four DENV serotypes [26]; properties which make it a possible therapeutic candidate for treatment of DENV infection.

Although an accelerated reduction of viral burden during acute dengue has been hypothesized to impact clinical outcome, this remains unproven. Failure of all clinical trials to date with small molecule-based therapeutics [27–29] in achieving *a priori* defined endpoints of dengue viral reduction may be due to underestimation of total viral burden, which can be as high as 10 logs of genome equivalents per ml of serum or plasma [27,29–31]. While the recently identified and characterized cross-serotype antibodies, have demonstrated strong anti-viral activity and draw attention to the potential for immunotherapy, several questions remain to be answered. Williams, *et al* reported that for some fusion loop and DIII directed antibodies, ability to suppress antibody-enhanced infection *in vitro*, and not their neutralization potency, was more predictive of *in vivo* therapeutic efficacy [32], suggesting that competition with endogenous antibodies may indeed significantly impact clinical potency of candidate therapeutic antibodies. Resistance development under immunological pressure is an additional concern. In this study, we conducted *in vitro*, *in vivo*, and *ex vivo* studies that examined the activity of VIS513, targeting domain III of the E protein, in reducing clinically relevant viral burden, with or without antibody-enhanced infection, as well as propensity for viral escape by all four dengue serotypes, to understand the potential as a therapeutic agent for dengue.

## Materials and methods

### Animal strains, virus strains, cell lines

A129 and AG129 breeders were imported from B&K Universal (UK). A colony each of A129 and AG129 mice was established at NUS Comparative Medicine under approved IACUC breeding protocol # BR12-28 and BR04-12, respectively. The mice were transferred to ABSL2 rooms when DENV infection was to be performed. DENV infections and mAb treatments are described in the approved IACUC protocol R13-04751. C6/36 (ATCC CRL-1660), BHK-21 (ATCC CCL-10), Vero (ATCC CCL-81) and THP-1 (ATCC TIB-202) cells were purchased from American Type Culture Collection (ATCC). THP-1.2S was subcloned from THP-1 by limiting dilution as previously described [33]. D2Y98P-PP1 is a DENV2 strain derived from a clinical isolate (Genbank #JF327392) from Singapore in 2000. DENV1 05K3903DK1 (Genbank #EU081242) was isolated from a patient during a DENV outbreak in Singapore in 2005 [34]. DENV2-MT5 is a viral strain derived from the D2Y98P-PP1 through a single amino acid substitution (Phe->Leu) at position 52 of the NS4B protein [35]. The DENV strains were propagated in the *Aedes albopictus* cell line C6/36 and maintained in Leibovitz’s L-15 medium (Gibco) supplemented with 10% fetal calf serum (Gibco). Virus propagation and harvest were carried out as described previously [36]. Virus stocks were stored at -80°C. Virus titers of stocks were determined by plaque assay in BHK-21 cells as described below. The viral strains used for escape analysis—DV2-NGC (Genbank accession KM204118.1), DV3-H87 (Genbank accession KU050695.1), DV1-Haw44 (Genbank accession KM204119.1) and DV4-BC287 (Genbank accession AF459627.1) were obtained from BEI resource repository (VA, USA) and propagated in BHK-21 cells for preparation of high titer stocks. Primary monocytes were isolated from an IRB-exempt donor and cultured as previously described [37].

### Virus neutralization and plaque assays

For VIS513 neutralization in the presence of competing serum, DENV was incubated with enhancing concentration of DENV-immune serum or similar dilution of naïve serum for 45 minutes at 37°C. Immune complex was then added to serial two-fold dilutions of VIS513 or its isotype control antibody CDA1, and incubated for 45 minutes at 37°C, before adding to THP-1.2S cells or primary monocytes (MOI of 10). The infectious titer of DENV in the culture supernatant was quantified with plaque assay at 72 hours post-infection. Percent neutralization was calculated using the following formula: Percent neutralization = [(virus only (pfu)–virus-antibody complex (pfu)) / virus only (pfu)] × 100. PRNT_50_ values were determined using a sigmoid dose-response curve fit in Prism 5.0.

Plaque assay was carried out in BHK-21 cells as described previously [36]. Briefly, 5x10^4^ BHK-21 cells were seeded in 24-well plates. BHK-21 monolayers were infected with 10-fold serially diluted viral suspensions ranging from 10^−1^ to 10^−4^. For the A129 mice experiments involving antibody administration at day 1, 2-fold dilutions, ranging from 40 to 160, were also employed (after determining from the first experiment that 10-fold dilutions resulted in lower sensitivity). After 1 hour incubation at 37°C with 5% CO_2_, 1% (w/v) carboxymethyl cellulose was added to the wells. After 4–5 days incubation at 37°C with 5% CO_2_, the cells were fixed with 4% paraformaldehyde and stained with 1% crystal violet. The plates were rinsed thoroughly, dried and the plaques were scored visually and expressed as the number of plaque forming units (PFU).

### Escape mutation analysis and structural modeling

High titer virus for all four serotypes of DENV was pre-mixed with varying concentrations of antibody which was predicted to neutralize the majority of the input virus, or trastuzumab (Herceptin) as a negative control. The virus-antibody mixture was pre-incubated for 1 hour at 37°C in propagation media (DMEM, 2% FBS, 1X non-essential amino acids, Glutamax and 20 mM HEPES). Non-neutralized virus was allowed to infect Vero cells for 2 hours at 37°C at which time the antibody/virus mixture was removed and replaced with 3 mL of fresh propagation media containing antibody at the same concentration. Infected Vero cells were incubated at 37°C with 5% CO2 for 72–96 hours to allow virus propagation to occur. At the end of the 72–96 hour incubation, the supernatant containing virus was collected and half the volume was again pre-mixed with varying amounts of antibody, thus repeating the selection and propagation process. This propagation process was performed for a total of seven passages for each virus and antibody combination. The negative control antibody, trastuzumab, was included to identify changes in virus sequence and neutralization potency arising independent of neutralization resistance (e.g., culture adaptation). At passages 5 and 7, aliquots of virus were frozen for plaque titer determination, micro-neutralization (MN) assays and subsequent sequencing of the virus.

Sequencing of all detectable viruses was performed by extracting viral RNA using the QIAamp Viral RNA Mini kit and performing RT-PCR with primers designed to amplify the entire E gene, from the prM to NS1 gene, using the One-Step Superscript III RT-PCR System with Platinum Taq. The resultant amplicon (~1,800 bp) was purified using a Qiagen PCR purification kit and sequenced.

For NGS studies, amplicons were generated in two separate PCR reactions to yield approximately 1 μg of purified cDNA which encoded the DENV1 or DENV2 E-gene. Reverse transcription PCR (RT-PCR) primers (DV2NGC-Forward TGGATGTCATCAGAAGGGGCCTGGAAACATGCCC; DV2NGC-Reverse CGGATTCCACAAATGCCCTCTTCATGAGCTTTCTGG) and subsequent nested PCR primers (DV2nestForward-CCAGAGAATTGAAACTTGGATCTTGAGACATCCAGGC; DV2nestReverse GAGCTTTCTGGATAGCTGAAGCTAGCTTTGAAGGGG) were designed in such a way that the entire DENV E-gene would be contained within the amplicon with additional 5’ and 3’ viral genome sequence to ensure full E-gene coverage with NGS. The RT-PCR was performed using a fresh aliquot of RNA using the SuperScript III One-Step RT-PCR System with Platinum Taq DNA Polymerase (Life Technologies Cat# 12574–026). A secondary nested PCR was set up using the purified RT-PCR product as the template for each reaction. NGS was performed by Genewiz on the Illumina MiSeq platform using a 2 x 150 bp paired end configuration. Sample data sets were mined and aligned against reference sequences from Sanger sequencing data generated using DENV1 and DENV2 reference strains. NGS data was manually curated to identify residues with changes with ≥ 5% prevalence when compared to the isotype control samples for a given experiment.

The crystal structures of monoclonal antibody 4E11 in complex with the domain III of the E protein of DENV1, DENV2, DENV3 and DENV4 proteins (PDB 3UZQ, 3UZV, 3UZE and 3UYP) and the 4E11-related mAb VIS513 in complex with DENV4 domain III E protein (PDB 5AAM) were used to analyze putative escape mutants. For this analysis, the VIS513-EDIII-DENV3 protein complex was generated by super positioning (only backbone atoms) of EDIII-DENV3 structure (PDB 3UZE) onto the EDIII-DENV4 protein of the VIS513-EDIII-DENV4 complex. The escape residues were modeled using PyMol software without energy minimization. 14C10 antibody bound DENV particle complex was analyzed using the coordinates (PDB 4CAU) generated from electron microscopy.

### EDIII purification and binding

For determining VIS513 affinity to EDIII mutants, the DNA encoding the EDIII domain of the E protein (amino acids 293–400) from multiple prototypic strains was synthesized (DNA2.0) in frame with the coding region for a (His)6 epitope tag and cloned into the bacterial expression vector pJexpress414. For the generation of DNA encoding mutant EDIII proteins, QuickChange mutagenesis (Agilent) was performed as described by the manufacturer on the appropriate wild-type EDIII vector. Origami 2 (DE3) E. coli (EMD Millipore) were transformed with the vector and transformant cultures were scaled to the desired volume and EDIII expression induced with 1mM IPTG for 3 hours at 20°C. Bacterial cells were harvested, cells disrupted by sonication, DNA removed with benzonase and EDIII purified from the cell lysate by immobilized metal affinity chromatography.

The affinities of antibodies (KD) to EDIII, in solution at equilibrium, were determined by competition ELISA [26,38]. Briefly, serial dilutions of EDIII were mixed with antibody at 0.2nM and mixtures were incubated overnight to allow equilibrium to be reached. An indirect EDIII ELISA was then performed to measure the concentration of unbound or singly bound antibody. Briefly, Maxisorp plates were coated with EDIII-DV1 and equilibrium antibody–EDIII mixtures were added to the wells. Bound antibody was detected by HRP-conjugated rabbit anti-human IgG (Jackson ImmunoResearch). The data were fit by least squares regression using a mass action model, with adjustment to take into account antibody bivalence [39].

### AG129 mouse model for primary dengue infection

In the primary infection model, eight to nine weeks old AG129 mice were infected via the subcutaneous (s.c) route with 10^6^ plaque forming units (PFU) of DENV1 or DENV2 viral strains. On day 2 post-infection, mice were administered various amounts of either control antibody (trastuzumab) or VIS513, diluted in sterile PBS, intravenously. Doses were adjusted based on the weight of the animals to ensure that the appropriate amount of therapeutic (in mg/kg) was delivered. At day 5 post-treatment, the mice were terminally bled and sera used in plaque assays to quantify viremia. Serum samples from animals treated with 5mg/kg or 25mg/kg of VIS513 were additionally used for NGS analysis.

### Secondary infection maternal enhancement model in A129 mice

The A129 model of maternal antibody mediated enhanced disease has been described previously [40]. Briefly, five to six week old female A129 mice were infected with 10^6^ PFU of DENV1 via the intravenous (iv) route (0.1 mL in sterile PBS) leading to asymptomatic infection. One week post-infection, after viral clearance, the females were allowed to mate with naïve 6-week old A129 males and resulting pups were weaned 21 days following birth. At 5–6 weeks of age, mice born to DENV1-immune mothers were administered 10^6^ PFU of DENV2 (D2Y98P-PP1) via the i.v. route (0.1 mL in sterile PBS). At day 1 or day 2 post-infection mice were administered various amounts of either control antibody (trastuzumab) or VIS513, diluted in sterile PBS, intravenously. Doses were adjusted based on the weight of the animals to ensure that the appropriate amount of therapeutic (in mg/kg) was delivered. Beginning on day 1, clinical symptoms were scored as follows: 0—healthy state, 1—signs of ruffled fur, 2—hunched back, 3 –severe diarrhea, 4—lethargy, 5—moribund. On day 4 post-infection (the time point which animals within the untreated and isotype antibody-treated groups were moribund) 4 mice per group were bled via the retro-orbital route. After allowing the blood to clot, the serum was collected by centrifugation and stored immediately at -80°C until performing the plaque assay (by performing 10-fold serial dilutions of the serum) in BHK-21 cells to determine viral titers (in terms of PFU/mL). An aliquot of serum from animals treated with 5mg/kg or 25mg/kg of VIS513 was used for the NGS analysis as described above. A separate group of animals for each treatment was monitored for survival and clinical score daily until the score reached 3 after which the animals were monitored at a frequency of two to three times a day. Survival rate was derived from the number of mice that were euthanized at moribund stage as evidenced by ruffled fur, hunched back, severe diarrhea, and extreme lethargy. Quantitation of genome copies in serum was performed using Superscript III Reverse Transcriptase (Life Technologies) followed by Taqman Universal Master Mix II (Life Technologies) with primers directed to the NS1 gene. To determine genome copy equivalents (GCE), a portion of the NGC NS1 gene was cloned into a plasmid, and a titration of plasmid with known input number of molecules was used in each experiment to generate a GCE standard curve. Additionally, cDNA from a sample of DENV-2 NGC virus stock of known titer was also analyzed and used to relate GCE to PFU/mL.

### Statistics

As indicated in figure legends, student t-test or 1 way ANOVA with Dunn’s multiple comparison test was used to determine statistical significance between various samples. All error bars show standard error of the mean (S.E.M.). All calculations were performed using GraphPad Prism v5.0 (GraphPad Software Inc.).

### Ethics statement

A129 and AG129 breeder mice were imported from B&K Universal (UK). Breeding and experimental procedures described in this work were carried out according to the guidelines of the National Advisory Committee for Laboratory Animal Research (NACLAR) in the AAALAC-accredited National University of Singapore (NUS) animal facilities (http://nus.edu.sg/iacuc/), and were approved by the NUS Institutional Animal Care and Use Committee (IACUC) under protocol numbers BR12-28 and BR04-12 (breeding protocol) and R13-04751 (experimental protocol). Non-terminal procedures were performed under anaesthesia, and all efforts were made to minimize suffering. Human sera used in this study including DENV1 05K3903DK1 (Genbank #EU081242) were obtained from the early dengue infection and control (EDEN) study as previously described [34] and approved by the National Healthcare Group Domain Specific Review Board (DSRB B/05/013). These anonymized samples were from adult patients aged 21 years and above, who provided written informed consent for the use of material and clinical information for research purposes.

### Accession numbers

Viral strains with the following accession numbers were used for experiments and analysis—DV2-NGC (Genbank accession KM204118.1), DV3-H87 (Genbank accession KU050695.1), DV1-Haw44 (Genbank accession KM204119.1), DV4-BC287 (Genbank accession AF459627.1), D2Y98P-PP1 (Genbank #JF327392) and DENV1 05K3903DK1 (Genbank #EU081242).

## Results

### Efficiency of cross-serotype neutralization of VIS513 under physiologically relevant conditions

VIS513 is a humanized antibody that has previously been shown to bind and neutralize all four DENV serotypes and abrogate multiple aspects of severe dengue infection, including cytokine elevation, thrombocytopenia, and vascular leak [26]. As a baseline to additional studies, we compared the neutralization potency of VIS513 to other broadly binding, neutralizing antibodies– 752–2 C8 and 747(4) A11, as the most potent members of the EDE antibodies (class 1 and 2, respectively) and 4G2, a fusion loop directed antibody. We find that the PRNT_50_ values for VIS513 are significantly lower than those for 4G2, comparable to those for 752–2 C8 and generally higher than those for 747(4) A11 (**Table 1**).

**Table 1:** PRNT_50_ (ng/mL) values for anti-DENV antibodies.

Antibody	DENV1	DENV2	DENV3	DENV4
**VIS513**	54.7	15.1	216.7	55.7
**752–2 C8**	22.6	84.3	347.6	255.3
**747(4) A11**	7.4	25.4	25.4	7.9
**4G2**	**560**	**10,700**	**2,400**	**2,300**

Neutralization potency of antibodies, frequently determined by the antibody titer that neutralizes 50% of the viral inoculum on a plaque reduction neutralization test (PRNT_50_), can be greatly influenced by host cell type and other assay conditions [41,42]. Moreover, DENV infection can be enhanced by cross-reactive antibodies that are present during secondary DENV infection. Indeed, *in vitro* neutralization potency of some antibodies has been shown to be significantly reduced in the presence of enhancing levels of antibodies found in mouse immune sera [32]. Additionally, neutralizing antibody titers required for protection in human cohorts are reported to be higher for serotypes such as DENV2 [43]. In the context of therapeutics, this may translate to a requirement for a higher dose of neutralizing antibody for therapeutic efficacy.

**Fig 1:**
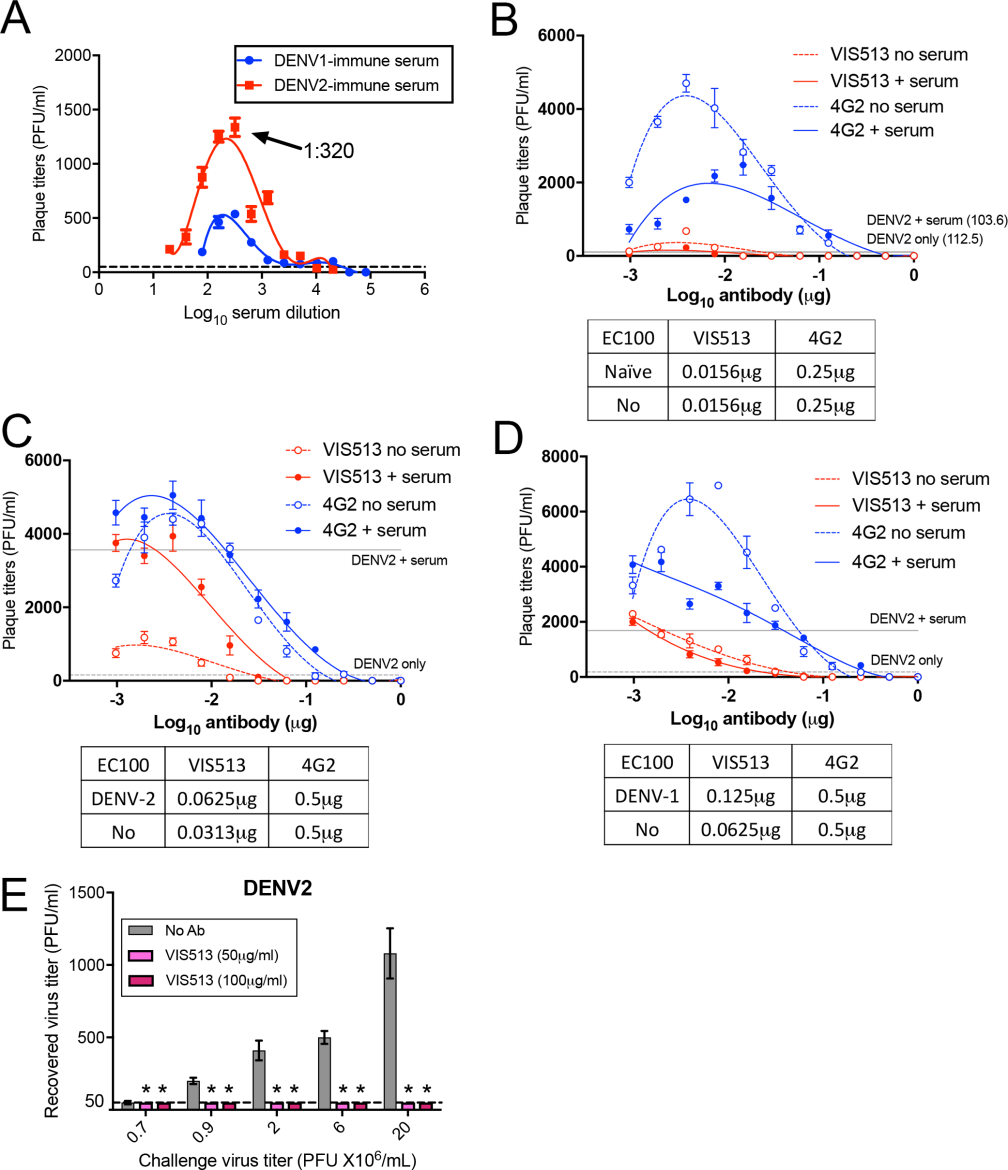
Neutralization of DENV by VIS513 under various conditions in vitro. A. Infectious titers of DENV2 in the culture supernatant of THP-1.2S incubated with DENV2 (moi 10) reacted with serial two-fold dilutions of convalescent primary DENV1 (blue) and DENV2 (red) sera. Serum dilution of 1:320 resulting in peak enhancement of DENV2 infectious titers is indicated by arrow. Dotted line indicates infectious titers following DENV2 only infection in THP-1.2S. B-D. Infectious titers of DENV2 in THP-1.2S cells following VIS513 or 4G2-mediated neutralization of DENV2 incubated with naïve serum at 1:320 dilution, or in the absence of serum (B). Infectious titers were also measured following VIS513 or 4G2-mediated neutralization of DENV2 incubated with convalescent primary DENV2 (C) and primary DENV1 serum (D) at 1:320 dilution, or in the absence of serum. Dotted lines represent infectious titers following DENV2 infection in the presence or absence of serum, when assay was performed in the absence of VIS513 or 4G2. E. Neutralization efficiency of 50μg/mL or 100μg/mL of VIS513 against different titers of DENV2. Virus incubated without antibody served as control. Dotted line indicates limit of detection for plaque assay. Data are represented as mean ± SEM from two independent experiments.

Given this background, we tested the ability of VIS513 to neutralize DENV2 in the presence of enhancing concentrations of convalescent serum obtained from patients with primary DENV1 or DENV2 infection. We first determined the dilution of convalescent primary DENV1 or DENV2 sera resulting in peak enhancement of DENV2 infection in the THP-1.2S monocytic cell line, a subclone of THP-1 that has been previously shown to be more susceptible to antibody-enhanced infection [33] (**Fig 1A**). Subsequently, a neutralization assay with VIS513 was performed against DENV2 incubated with peak enhancing dilutions (1:320) of DENV2 immune or a matching dilution of naïve sera, or DENV2 in the absence of sera.

Complete DENV neutralization mediated by VIS513 remained unaffected in the presence of dengue-naïve serum (**Fig 1B**), DENV2 immune serum (**Fig 1C**) and DENV-1 immune serum (**Fig 1D**) relative to infection in the absence of serum. This is reflected in EC100 values for VIS513 that were either identical or only showed 2-fold difference between conditions in the absence or presence of serum. We also included 4G2, a fusion loop-targeting antibody, for comparison in this experiment (**Fig 1B–1D**). While EC100 values for 4G2 also remained identical in the absence or presence of serum, higher amounts of 4G2 relative to VIS513 were required for complete DENV neutralization. In the presence of naïve or DENV-immune serum, less VIS513 (EC100_naive_ 0.0156 μg, EC100_DENV1_ 0.125 μg, EC100_DENV2_ 0.0625 μg) relative to 4G2 (EC100_naive_ 0.25 μg, EC100_DENV1_ 0.5 μg, EC100_DENV2_ 0.5 μg) was required for complete DENV neutralization.

Neutralization curves were also plotted for VIS513 and 4G2 under these conditions (**S1 Fig**) and was similar to neutralization curves plotted when the assay was performed using primary monocytes (**S1G Fig**), suggesting this was not a cell type-specific event. Neutralization curves plotted for VIS513 and 4G2 showed a similar trend to infectious titers following neutralization with either VIS513 or 4G2 (**Fig 1B–1D**). EC50 inferred from the neutralization plots were also concordant with antibody concentrations required for complete neutralization (EC100) in THP-1.2S cells, with higher EC50 observed for 4G2 relative to VIS513 (**S1 Fig**). Taken together, the data suggest that VIS513 retains its neutralization potency and suppresses DENV2 virus infection in a background of enhancing antibodies. Notably, these data suggest that a higher dose of VIS513 is not likely required to treat secondary compared to primary dengue.

Dengue in humans is characterized by variable peak viral titers that can span five orders of magnitude, from 10^5^−10^10^ genome equivalents/mL [29], corresponding to highest levels of peak infectious particles of 10^7^−10^8^, assuming a non-infectious to infectious particle ratio of 1:100–1000 [44]. This viral burden is in sharp contrast to the commonly used plaque reduction assay to estimate EC_50_, which often uses only 50–100 infectious particles in any single reaction. To determine the ability of VIS513 to neutralize viral loads that are clinically relevant, we titrated differing amounts of virus against a fixed concentration of antibody. Varying titers of DENV2 were incubated with VIS513 at either 50 μg/mL or 100 μg/mL before addition to THP-1.2S cells. These concentrations of VIS513 were chosen since intravenous administration of weight-adjusted doses of antibody from 2–4 mg/kg in humans has been shown to achieve serum C_max_ levels of approximately 50–100 μg/mL, thus representing an easily achievable circulating level of antibody [45]. After the virus/antibody mixture was incubated with THP-1.2S cells for 72 hours, progeny virus was harvested from supernatants and viral titer determined by plaque assay. VIS513 at 50 μg/mL or 100 μg/mL completely neutralized DENV2 when present at up to 2x10^7^ PFU/mL (2.56x10^8^ genome copies/mL, MOI of 100), which was the highest viral load that could be tested in this assay due to constraints in virus stock preparation (**Fig 1E**). A similar experiment testing neutralization of other serotypes could not be performed due to constraints in obtaining high virus titers from clinical isolates.

### VIS513 efficacy in mouse models of DENV infection

**Fig 2:**
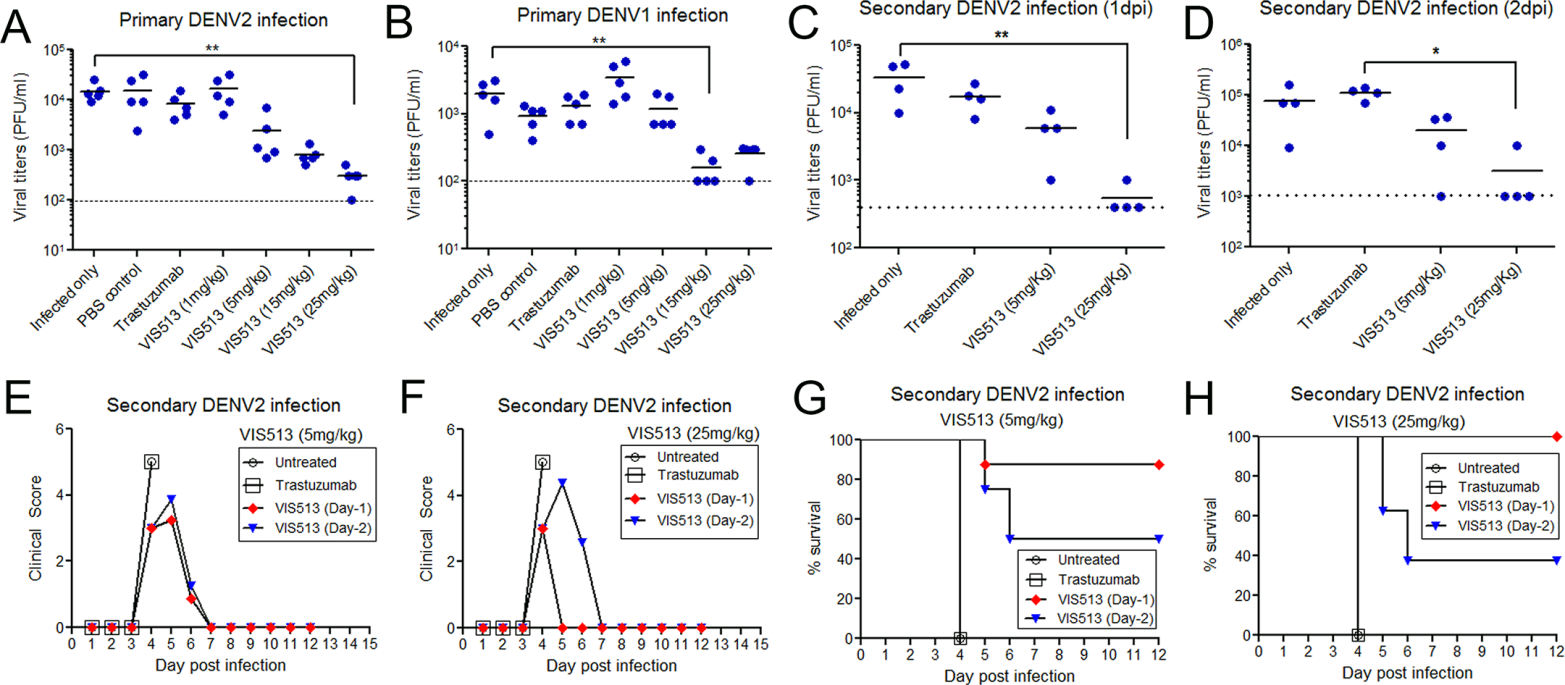
VIS513 treatment upon onset of viremia reduces viral titers in mouse models of primary or secondary dengue infection. A-B. Serum viral titers on day 5 post infection in a primary infection model of AG129 mice infected with DENV2 (A) or DENV1 (B) and treated at day 2 post infection (n = 5 per group). C-D. Serum viral titers on day 4 in a maternal antibody-mediated disease enhancement model in A129 mice upon treatment as indicated (n = 4 per group) at day 1 (C) or day 2 (D) post infection. **<0.01, * p<0.05 based on 1 way ANOVA with Dunn’s multiple comparison test. Dotted lines in A-D represent the limit of detection of the plaque assay. E-F. Impact of VIS513 treatment at 5mg/kg (E) or 25mg/kg (F) on morbidity in a lethal enhancement model in A129 mice. Symbols represent mean of data from 7–8 animals per group. G-H. Impact of VIS513 treatment at 5mg/kg (G) or 25mg/kg (H) on survival in a lethal enhancement model in A129 mice. Symbols represent mean of data from 7–8 animals per group. A version of the data from Day-1 administration in panels E-H has been published previously [26] and is included here for comparison.

To determine if the efficacy observed *in vitro* would extend to *in vivo* infection, we tested the efficiency of viral reduction by VIS513 administered therapeutically in primary and secondary infection with previously described AG129 and A129 mouse models, respectively. To assess primary infection, we infected AG129 mice subcutaneously with a clinical isolate of DENV2 or DENV1 and at 2 days post-infection (d.p.i.) animals were either untreated or treated with vehicle control, an isotype control antibody (25 mg/kg) or VIS513 at doses of 1, 5, 15 or 25 mg/kg. At 5 d.p.i., which corresponds to peak viremia in this model, all mice were terminally bled, and viral titers in sera were determined. There was no statistically significant difference in viral titers between the untreated animals, animals administered vehicle only, or animals administered the isotype control antibody. In all animals treated with 1 mg/kg of VIS513, there was no difference in viral titers compared to the various control groups. Treatment with 25 mg/kg VIS513 resulted in significant viral reduction of DENV2, with 1.7 log_10_ reduction of viral titers (**Fig 2A**). This experiment was repeated using a clinical isolate of DENV1 and treatment with 15 mg/kg of VIS513 resulted in significant reduction of viral titers (**Fig 2B**). In DENV1 infected mice treated with 1 mg/kg of VIS513, we observed a slight elevation in mean viremia compared with isotype control group that was not statistically significant. The possibility of this being an ADE mediated increase in viremia remains to be explored. Notably, this asymptomatic transient viremia animal model could only be established for DENV1 and DENV2, and as such prevented similar evaluation of DENV3 and DENV4.

Antibody-enhanced dengue infection has been recapitulated in several mouse models based on administration of sub-neutralizing levels of antibody, passive transfer of immune sera, or maternal antibody transfer [40,46,47]. To better elaborate the levels of viral reduction requirement to mediate protection, we tested the ability of VIS513 to reduce viral loads in an A129 mouse model of maternal antibody-mediated enhanced lethal disease [40]. In this model, pups born to DENV1 immune A129 mice develop lethal infection when infected with a sub-lethal dose of a DENV2 strain while those born to DENV-naïve mothers remain predominantly asymptomatic. In this model, VIS513 when administered at either 1 or 2 d.p.i at 5 mg/kg or 25 mg/kg reduced viral titers as measured at 4 d.p.i. (**Fig 2C and 2D**). Consistent with our previous observations [26], clinical scores and survival analysis of a separate group of mice treated with VIS513 either at 1 or 2 d.p.i. also revealed significant reduction in morbidity (**Fig 2E and 2F**) as well as mortality (**Fig 2G and 2H**) with greater protection seen when VIS513 was administered at 1 d.p.i. Collectively, these results demonstrate that in this lethal model of infection, VIS513 is able to impact DENV2 viral titers (as well as morbidity and mortality) to an extent sufficient to mediate partial or complete protection in a dose and time dependent manner.

### Analysis of serotype specific escape mutations leading to VIS513 resistance

**Fig 3:**
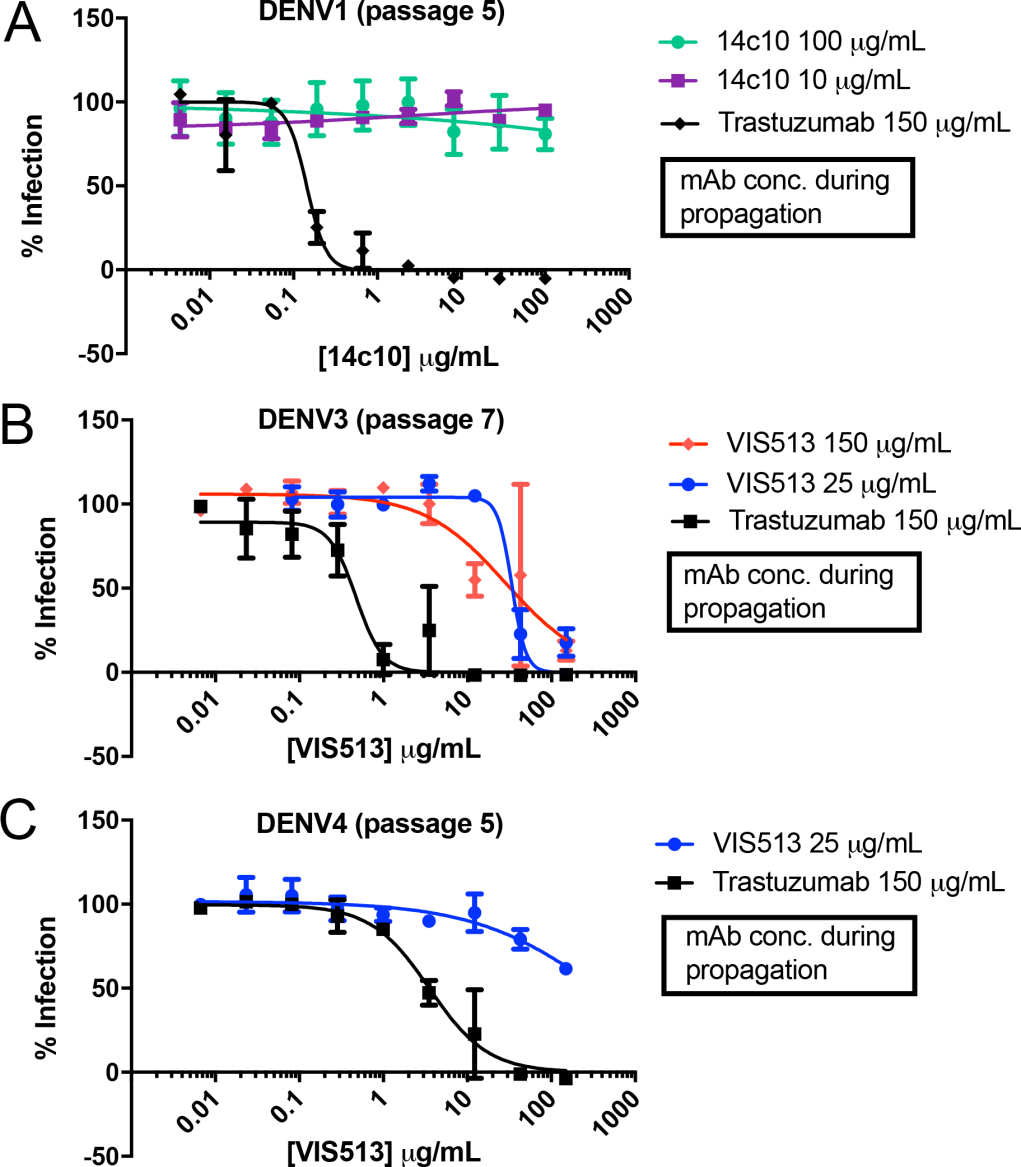
Identification of VIS513 escape mutants. A. Neutralization profile of a titration of 14c10 with DENV1 population passaged in presence of 14c10 at 100 μg/ml, 10 μg/ml, or trastuzumab at 150 μg/ml for 5 passages. B-C. Neutralization profile of a titration of VIS513 with DENV3 (passage 7) (B) or DENV4 (passage 5) (C) passaged in the presence of indicated concentrations of VIS513 or trastuzumab. For DENV4, passaging in the presence of 150 μg/ml VIS513 yielded insufficient virus for subsequent neutralization assays and hence could not be assayed. The solid lines and error bars represent mean ± SEM of experiment performed in triplicate.

A major challenge for any therapeutic against DENV (or any RNA virus) is whether the anti-viral activity induces or selects for resistance. Neutralizing responses to DENV infection in humans include the induction of antibodies to quaternary epitopes near the hinge region of DI/DII that are highly potent but serotype specific, and are susceptible to viral escape [11,48]. To directly examine viral escape to a DIII-directed antibody, we serially passaged DENV strains representative of serotypes 1–4 in the presence of fixed amounts of VIS513 or an isotype control. For reference, we also passaged DENV1 virus in presence of 14c10, a previously identified, highly potent, DENV1-specific human antibody for which escape mutations have been described [11,48]. For VIS513, no viral escape was detected with DENV1 or DENV2 up to seven serial passages with either 150 μg/mL or 25 μg/mL of VIS513. In contrast, virus passaging in the presence of 14c10 resulted in viral escape of DENV1, with emergence of resistance at passage 5 and virus completely resistant to neutralization by passage 7 (**Fig 3A**).

For DENV3, viral escape from VIS513 was detected at passage seven, while for DENV4 viral escape was detected at passage five. Unlike for 14c10, analysis of the DENV3 and DENV4 escape population indicated reduced, but not fully ablated, neutralization by VIS513 (**Fig 3B and 3C**). These results indicate that in contrast to 14c10, the highly conserved epitope engaged by VIS513 is less mutable. In support of this reasoning, mutations in EDIII that lead to reduction of antibody neutralization have been previously noted to be important residues for viral fitness (see below). Additionally, with the related West Nile virus, it has been noted that resistance development to a DIII-directed antibody *in vivo* is delayed and, while such resistance effects *in vitro* neutralization, *in vivo* protection is not substantially effected [49].

**Fig 4:**
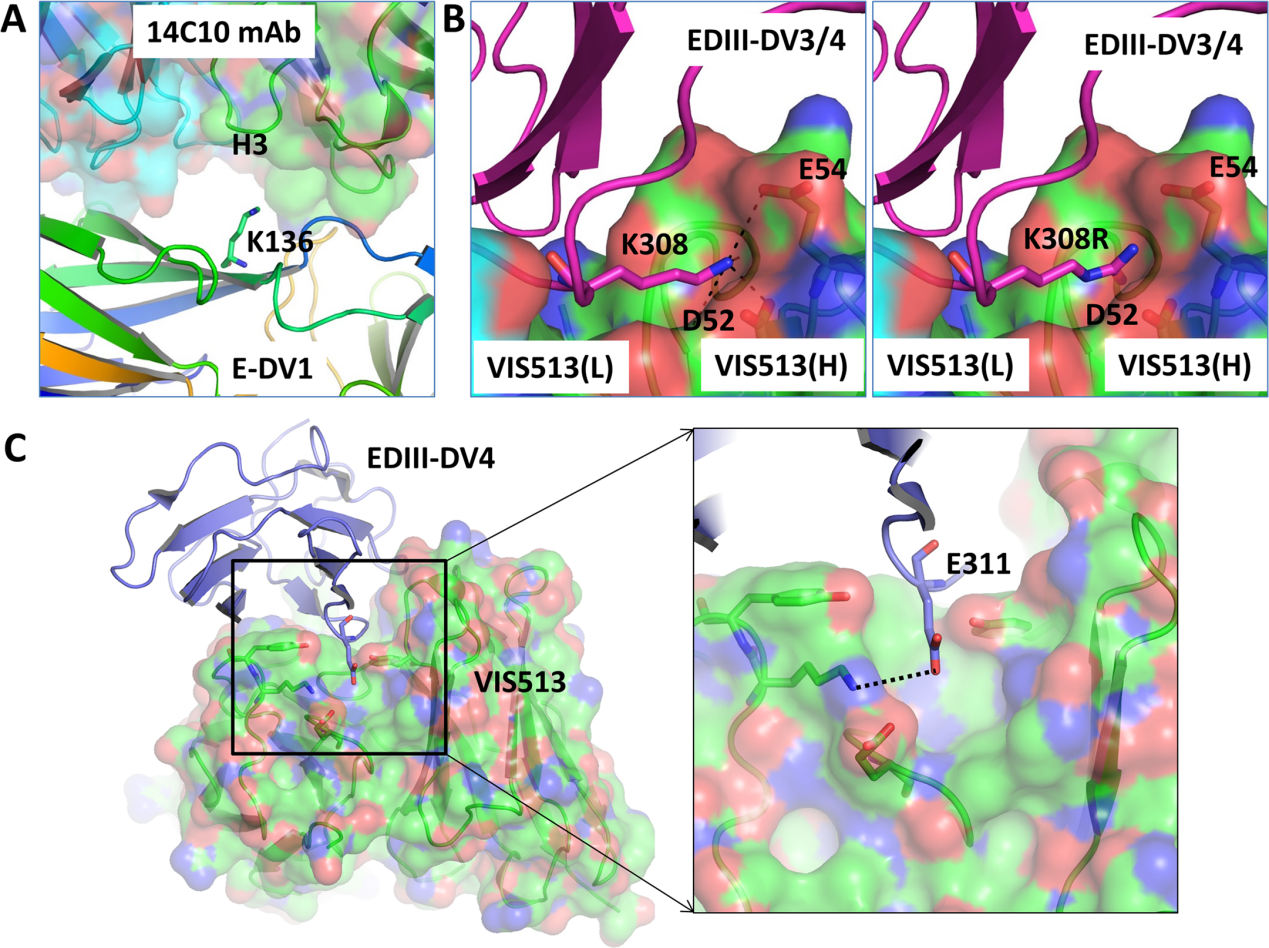
Structural analysis of escape mutations. A. Molecular interactions of E-DENV1 protein (shown in rainbow color cartoon) are shown with the 14c10 antibody (shown in transparent surface and cartoon diagram with heavy and light chains shown in green and cyan color, respectively). In the complex, the Lys136 residue of the E-DENV1 protein is found in the vicinity of the HCDR3 loop of the mAb. Mutation of Lys136 to a Glu is expected to cause loss of mAb binding due to the change in charge of the residue. B. Molecular interactions of Lys308/310 of EDIII-DENV3/4 protein (shown in magenta cartoon) are shown with VIS513 (shown in transparent surface and cartoon diagram with heavy and light chains shown in green and cyan color, respectively). The Lys308 of E-DENV3 or Lys310 of E-DENV4 residues are the same and it is found to form hydrogen bonding and salt bridges with the VIS513 heavy chain residues Asp52 and Glu54 (left panel). The right panel shows the modeling of mutation Lys308Arg. C. Modeling of position E311 of E-DENV4 region found to be mutated in virus from mouse samples. A hydrogen bond with the antibody molecule is shown with dotted line.

We next determined the sequence of the envelope gene of viruses passaged in the presence of 14c10 (DENV1), VIS513 (DENV3 and DENV4) or isotype control to identify the sites of potential escape (**Table 2**). The DENV1 virus passaged in the presence of 14c10 revealed Lys136Glu and Lys204Arg mutations in two independent samples relative to virus passaged in the presence of isotype control. The Lys136Glu mutation has been reported to be part of the 14c10 epitope [11]. Examination of published cryoelectron microscopy structures of 14c10 Fab complexed with DENV1 E protein revealed that the Lys136Glu mutation is within the DI/DII hinge region and specifically within the epitope footprint of 14c10 (**Fig 4A**). We did not identify the previously reported Thr51Lys ecape mutation which could be attributed to differences in methods and/or the virus strain used in the studies [48]. Of note, the Lys204Arg position is unlikely to be part of the 14c10 epitope and this amino acid change does not alter the overall charge (Lys→Arg). One possibility for the emergence of this change under selective pressure with 14c10 is that position 204 in the DENV1 E protein has been identified as important for virus envelope “breathing”, thereby indirectly affecting epitope accessibility and hence antibody binding [50].

**Table 2:** Summary of escape mutations observed from cell culture studies.

Serotype	mAb	Mutations (E gene)	Binding Affinity (nM)
DENV1 HAW	14c10	K136E K204R	ND ND
	VIS513	K310R WT	0.39 <0.1
DENV2 NGC	VIS513	K310R WT	0.79 <0.1
DENV3 H87	VIS513	L301S K308R WT	1.7 169.8 1.5
DENV4 BC287	VIS513	K310R WT	617.6 6.1

ND = not determined

Sequencing of DENV3 passaged in the presence of VIS513 revealed a Lys308Arg relative to the isotype control-treated sample (positions 310 by DENV1, 2, 4 numbering). An additional mutation, Leu301Ser, was identified in the VIS513 150 μg/mL sample relative to control virus (position 303 by DENV1, 2, 4 numbering). Interestingly, VIS513-propagated DENV4 virus revealed a single mutation Lys310Arg (residue 308 by DENV3 numbering), an identical mutation as one identified for DENV3. We structurally modeled the Lys308/310Arg (**Fig 4B**) mutation onto the co-crystal structure of the DENV3-EDIII complex [51] with 4E11 (precursor antibody to VIS513) to determine whether the mutations were located within the epitope region of VIS513. Lys308/310 is within the mAb epitope region and modeling of Arg instead of Lys suggests a potential steric clash of the Arg residue with CDR H1 loop (**Fig 4B, right panel**).

The impact of Lys308Arg and Leu301Ser mutations were further investigated by ELISA-based binding assays. Consistent with our structural analysis, the Leu301Ser mutation had no impact on binding (K_D_ of 1.7 nM relative to WT of 1.5 nM); however, as predicted by our *in silico* modeling, the Lys308Arg mutation significantly reduced binding (K_D_ of 169.8 nM as compared to 1.5 nM for WT), indicating that this mutation was the most likely determinant of viral escape for DENV3 and DENV4 (**Table 2**). When the Lys310 position was mutated to Arg in DENV1- or DENV2-derived E protein, only a minor decrease in VIS513 affinity was observed (**Table 2**), consistent with this mutation not arising when these serotypes were passaged under VIS513 selective pressure.

We also evaluated E gene sequence diversity in virus populations present from VIS513-treated A129 mice from the antibody enhanced disease model, as well as AG129 mice from the primary infection model using next generation sequencing. In nine animals challenged with DENV2 and treated with VIS513, no alterations were observed in the E protein that could be attributed to VIS513 administration. In DENV2-infected mice treated with 5 mg/kg VIS513, 1 of 9 animals demonstrated a mutation at position 311 (Glu311Gly) in 6.9% of the sequencing reads. In mice treated with a higher dose of 25 mg/kg VIS513 and challenged with DENV2, mutation at position 311 (Glu311Asp) was also observed in 1 of 9 animals (21% of sequencing reads). The 311 residue is highly conserved in E protein of all DENV serotypes. Structural analysis indicates mutation of Glu311 residue to an Asp or Gly residue may disrupt hydrogen bonding and van der Waals interactions resulting in a decrease in binding to VIS513 (**Fig 4C**). We note that this residue is also adjacent to the K308/310 residue which was identified as a site for escape mutation (in the context of DENV3 and DENV4, but not DENV1 or DENV2) as noted above. Also of note, neither of the two animals that developed low levels of putative VIS513 escape mutations showed viral kinetics suggestive of VIS513 efficacy failure. In fact, these animals had comparable viral load reductions to other animals that did not have virus with the E311 mutation.

To understand the prevalence in natural strains of variants at position 308/310 identified in the *in vitro* escape analysis, we analyzed all DENV strains reported in GenBank. The results indicated that Lys was present at this position in 99.7% or more of all strains from DENV1-3 and in 99.1% of strains from DENV4 (**Table 3**). Only Arg was observed as a polymorphism at this position in both DENV3 and DENV4, which was extremely rare with a frequency of <0.9%. These results demonstrate that position 308/310 is highly conserved, with very few strains that are predicted to have reduced sensitivity to VIS513 based on position 308/310 amino acid composition suggesting that overall propensity for viral escape to the VIS513 epitope was extremely low.

**Table 3:** Prevalence of alterations at position 310, or equivalent, in DENV1-4.

	DENV1			DENV2					DENV3		DENV4	
Position 310	K	R	E	K	R	E	M	W	K	R	K	R
Prevalence [%]	**99.89**	0.09	0.03	**99.7**	0.07	0.1	0.03	0.07	**99.94**	0.06	**99.1**	0.9
Seqs (n)	*3512*			*3054*					*1656*		*855*	

## Discussion

An emerging consensus from studies examining the humoral response to DENV infection indicates that the majority of the human response is targeted to the prM protein or to specific epitopes of the E protein [10,52]. From these studies, most of the cross-neutralizing monoclonal antibodies either bind complex quaternary epitopes [10] or within or near to the fusion loop of the E protein [12]. In contrast, the majority of antibodies targeting domain III of the E protein have specificities limited to DENV2 (*e*.*g*., 9F5), DENV1 (*e*.*g*., E106) [53], or DENV1-3 (*e*.*g*., 87–1) with the exception of VIS513 that exhibits pan-serotype neutralizing activity. The higher potency of the EDE antibodies, and VIS513, likely has to do with better epitope accessibility compared to the relatively inaccessible fusion loop targeted by 4G2. Characterization of the human antibody repertoire to DENV infection indicates that most antibodies directed to DI/II and the fusion loop are weakly neutralizing, and possess significant enhancing activity [12,16,17]. Within this context, polyclonal antibody potency is likely significantly impacted by the presence of competing antibodies that potentially block or shield neutralizing epitopes and/or parallel or competing enhancement of viral replication by enhancing antibodies. Given these characteristics of the endogenous antibody response and the attributes of VIS513, we evaluated its anti-viral activity under conditions of enhanced infection both in cell culture models as well as in animals.

We observed that VIS513- and 4G2-mediated neutralization of DENV2 *in vitro* was unaffected in the presence of enhancing concentrations of human dengue-immune sera across multiple serotypes. However, higher concentrations of 4G2 are required for complete neutralization of DENV, and this could be attributed to the distinct mechanisms of neutralization mediated by VIS513 and 4G2. While EC50 and EC100 values were inferred for the *in vitro* neutralization assays, EC100 values were used to compare neutralization efficiency of VIS513 and 4G2 as a therapeutic antibody would not be dosed at sub-neutralizing concentrations. Furthermore, we had chosen to perform the neutralization assay in THP-1.2S cells, as monocytes are one of the main targets of dengue infection in vivo. Critically, THP-1.2S cells express the full repertoire of Fc-gamma receptors, and allowed us to evaluate neutralization in the context of antibody-dependent enhancement (ADE). However, THP-1.2S cells produce significantly lower plaque forming virus in the absence of enhancing serum (Fig 1A). As the plaque titer for “no serum” or “naïve serum” condition is eventually used to calculate percent neutralization, the low values typically encountered under this condition suggest that any condition that results in a higher plaque titer is deemed “not neutralized”, which accounts for the steep neutralization plots seen for the “no serum” or “naïve serum” conditions (S1 Fig). Consequently, EC100 values would be more meaningful for assessing neutralization in THP-1.2S cells by a therapeutic antibody, as the objective is to mediate complete neutralization, or risk antibody-dependent enhancement especially if the antibody only mediates neutralization at the cell surface.

Indeed, the mechanism for neutralization is distinct for both antibodies and we have previously observed that VIS513, unlike 4G2, was able to neutralize DENV even when antibody-opsonized virions were phagocytosed via Fcγ receptors [26]. Conversely, high concentrations of 4G2 typically aggregate DENV, forming DENV immune complexes that cross-link the inhibitory FcγRIIB, which inhibits DENV uptake.

That VIS513 is able to neutralize DENV despite FcγR-mediated uptake of DENV immune complexes, suggests that VIS513 may tilt the threshold of enhancement to promote viral neutralization in a background of circulating antibodies. However, because both 4G2 and VIS513 were unaffected by enhancing sera, it is more likely that VIS513 efficiently displaces enhancing antibodies from virus, as previously demonstrated for antibodies such as E60 (DENV1-4, fusion loop) and E87.1 (DENV1-3, DIII), where this activity was correlated to their protection of mice in a lethal enhanced infection [32]. We corroborated this by demonstrating 100% survival of AG129 mice when they were treated 1 day post-infection with 25mg/kg of VIS513 (**Fig 2H**). Furthermore, others have shown that administering highly neutralizing concentrations of 4G2 that were protective *in vitro*, still led to lethality in AG129 mice [45].

In humans, DENV reaches high viral titers of up to 10^10^ genome equivalents per mL of serum [27,30] which is several orders of magnitude higher than that achieved in preclinical animal models. Therefore, we evaluated the ability of VIS513 at a fixed concentration to neutralize increasing amounts of virus in a clinically relevant range. At concentrations of 100 μg/mL, VIS513 was able to neutralize >10^7^ PFU, or >10^8^ genomic copies/mL of DENV2.

While neutralizing antibodies have been considered to be important for protection in dengue, there is limited data establishing clear correlation between *in vitro* potency and *in vivo* efficacy. We evaluated the potency of VIS513 in reducing viremia in AG129 mouse models of primary DENV1 or DENV2 infection where VIS513 reduced viral titers by >1Log_10_. However the extent of reduction was much lower than that predicted by *in vitro* studies likely due to ongoing viral replication in extravascular sites. Because mice in our primary infection model remained asymptomatic, it was not possible to correlate the viral reduction achieved by VIS513 with pathology. We therefore evaluated the efficacy of VIS513 in a lethal maternal antibody enhanced infection model. Single administration of VIS513 at 1 or 2 days post infection at 25mg/kg reduced mean viral loads by >1Log_10_ which was sufficient to reduce morbidity and mediate complete protection from mortality. Based on this, we predict that a goal of viral reduction by >1 log_10_ across all serotypes in a clinical setting, in both primary and secondary dengue infection, is likely to be achievable via administration of VIS513. Assessment of whether this level of viral reduction in humans is sufficient to impact disease severity can only be obtained through clinical studies. Additionally, the possibility and risk of enhanced viral titers at sub-neutralizing concentrations of therapeutic antibodies such as VIS513 due to ADE will need to be addressed through appropriate dose selection and monitoring.

Domain III of the flavivirus E protein has been suggested to have an important role in binding to cell surface receptors. Mutations in EDIII have also been associated with attenuation of virulence in dengue [54] as well as in other flaviviruses, such as yellow fever virus and tick-borne encephalitis [55,56], suggesting a fitness disadvantage for evolution or escape in this region. Additional factors could determine the impact of an escape mutation detected from *in vitro* studies or infection *in vivo*. For example, mice were protected by mAb E106 from lethal challenge by mutant DENV1 virus T329A, despite this mutation conferring resistance to neutralization by E106 *in vitro* [57]. While it remains to be investigated how mutations in the 308/310 position identified in our study (for DENV3 and DENV4) may directly impact viral fitness, a detailed phylogenetic analysis indicates that changes at this position are extremely rare for all four serotypes. Taken together, the data presented here suggest that targeting a non-immunodominant epitope in domain III represents a viable strategy to attenuate DENV during acute primary or secondary infection.

## Supporting information

S1 Fig Neutralization efficiency of 4G2 under various conditions. A-F. Neutralization of DENV2 by VIS513 (A-C) or 4G2 (D-F) in THP-1.2S cells following incubation of DENV2 either with dengue-naïve serum (at 1:320 dilution), or in the absence of serum (A, D). Neutralization of DENV2 by VIS513 and 4G2 was also assessed following incubation of DENV with peak enhancing dilutions of convalescent primary DENV2 (B, E) and primary DENV1 serum (C, F), or in the absence of serum. G. Neutralization of DENV2 by VIS513 in primary monocytes following incubation of DENV2 either with peak enhancing dilution of convalescent primary DENV1 serum, or in the absence of serum. The experiment was conducted in triplicates, data represented as mean ± SEM from two independent experiments. Dotted lines represent the mean background impact on viral replication when assay was performed in the presence of an isotype control antibody.
(TIF)
