# Supplementary figures and images for: Neutralization of antibody-enhanced dengue infection by VIS513, a pan serotype reactive monoclonal antibody targeting domain III of the dengue E protein
Source: PLoS Negl Trop Dis. 2018 Feb 9;12(2):e0006209. doi: 10.1371/journal.pntd.0006209 (PMC5823465; doi:10.1371/journal.pntd.0006209)

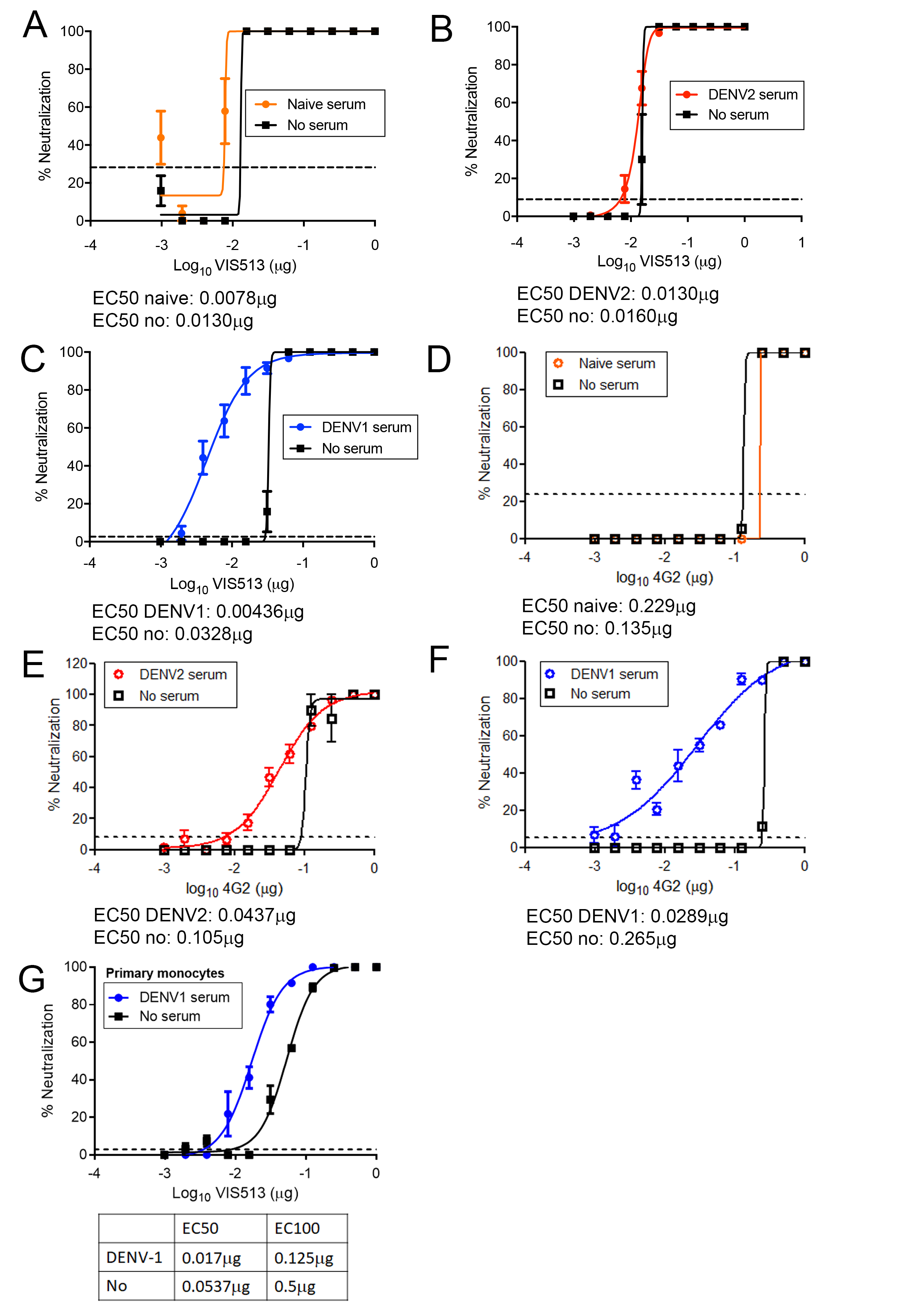

Supplement: S1 Fig — A-F. Neutralization of DENV2 by VIS513 (A-C) or 4G2 (D-F) in THP-1.2S cells following incubation of DENV2 either with dengue-naïve serum (at 1:320 dilution), or in the absence of serum (A, D). Neutralization of DENV2 by VIS513 and 4G2 was also assessed following incubation of DENV with peak enhancing dilutions of convalescent primary DENV2 (B, E) and primary DENV1 serum (C, F), or in the absence of serum. G. Neutralization of DENV2 by VIS513 in primary monocytes following incubation of DENV2 either with peak enhancing dilution of convalescent primary DENV1 serum, or in the absence of serum. The experiment was conducted in triplicates, data represented as mean ± SEM from two independent experiments. Dotted lines represent the mean background impact on viral replication when assay was performed in the presence of an isotype control antibody. (TIF) [file pntd.0006209.s001.tif]
